# Assessment of subchondral bone marrow lesions in knee osteoarthritis by MRI: a comparison of fluid sensitive and contrast enhanced sequences

**DOI:** 10.1186/s12891-016-1336-9

**Published:** 2016-11-16

**Authors:** Flemming K. Nielsen, Niels Egund, Anette Jørgensen, David A. Peters, Anne Grethe Jurik

**Affiliations:** 1 Department of Radiology, Aarhus University Hospital, Noerrebrogade 44, 8000 Aarhus, Denmark; 2 Department of Rheumatology, Aarhus University Hospital, Noerrebrogade 44, 8000 Aarhus, Denmark; 3 Department of Biomedical Engineering, Aarhus University Hospital, Noerrebrogade 44, 8000 Aarhus, Denmark

**Keywords:** Magnetic resonance imaging, Knee osteoarthritis, Bone marrow lesion, DCE-MRI

## Abstract

**Background:**

Bone marrow lesions (BMLs) in knee osteoarthritis (OA) can be assessed using fluid sensitive and contrast enhanced sequences. The association between BMLs and symptoms has been investigated in several studies but only using fluid sensitive sequences. Our aims were to assess BMLs by contrast enhanced MRI sequences in comparison with a fluid sensitive STIR sequence using two different segmentation methods and to analyze the association between the MR findings and disability and pain.

**Methods:**

Twenty-two patients (mean age 61 years, range 41–79 years) with medial femoro-tibial knee OA obtained MRI and filled out a WOMAC questionnaire at baseline and follow-up (median interval of 334 days). STIR, dynamic contrast enhanced-MRI (DCE-MRI) and fat saturated T1 post-contrast (T1 CE FS) MRI sequences were obtained. All STIR and T1 CE FS sequences were assessed independently by two readers for STIR-BMLs and contrast enhancing areas of BMLs (CEA-BMLs) using manual segmentation and computer assisted segmentation, and the measurements were compared. DCE-MRIs were assessed for the relative distribution of voxels with an inflammatory enhancement pattern, N_voxel_, in the bone marrow. All findings were compared to WOMAC scores, including pain and overall symptoms, and changes from baseline to follow-up were analyzed.

**Results:**

The average volume of CEA-BML was smaller than the STIR-BML volume by manual segmentation. The opposite was found for computer assisted segmentation where the average CEA-BML volume was larger than the STIR-BML volume. The contradictory finding by computer assisted segmentation was partly caused by a number of outliers with an apparent generally increased signal intensity in the anterior parts of the femoral condyle and tibial plateau causing an overestimation of the CEA-BML volume.

Both CEA-BML, STIR-BML and N_voxel_ were significantly correlated with symptoms and to a similar degree. A significant reduction in total WOMAC score was seen at follow-up, but no significant changes were observed for either CEA-BML, STIR-BML or N_voxel_.

**Conclusions:**

Neither the degree nor the volume of contrast enhancement in BMLs seems to add any clinical information compared to BMLs visualized by fluid sensitive sequences. Manual segmentation may be needed to obtain valid CEA-BML measurements.

**Electronic supplementary material:**

The online version of this article (doi:10.1186/s12891-016-1336-9) contains supplementary material, which is available to authorized users.

## Background

Pain associated with osteoarthritis (OA) of the knee is one of the main causes for disability in ageing Western populations [1, 2]. Although intensively studied, the pathophysiology and pain-causing mechanism in knee OA are generally unknown [3]. Recent literature evidence has shown that bone marrow lesions (BMLs), a non-specific but common feature in knee OA, may or may not be associated with pain [4–6]. There is also limited and conflicting evidence on pain severity being correlated to BML size or not [7–9].

BMLs are defined as poorly delineated areas of increased signal intensity directly adjacent to the subchondral bone in the normally fatty epiphyseal marrow on fat-suppressed T2-weighted and contrast enhanced T1-weighted images [10, 11]. On non-contrast MRI, BMLs are optimally evaluated using fluid-sensitive fat-suppressed sequences [12]. Histopathologically, BMLs represent a mixture of bone marrow edema, necrosis and fibrosis, microfractures with bleeding in different stages of healing, and remodeled trabeculae as well as fibrovascular ingrowth [13–15]. The increased vascularity explains why areas with BMLs show enhancement on static contrast enhanced (CE) T1-weighted images, preferably with fat saturation (T1 CE FS) [11, 16, 17], and dynamic CE MRI (DCE-MRI) sequences [18–20]. The volumetric proportion of detectable enhancement in knee OA using T1 CE FS has been reported to constitute 62% of the BML volume using proton density (PD) weighted FS sequences [11]. Comparison with DCE-MRI of BMLs has to our knowledge not been performed, although knee OA has been investigated with regard to synovial changes using DCE-MRI [21–23]. The diagnostic value of T1 CE FS and DCE-MRI compared to a STIR sequence only needs to be investigated in relation to BMLs. The same applies to a possible relation between contrast enhancing areas of BMLs (CEA-BMLs) and OA symptoms.

The aims of our study were to assess BMLs by contrast enhanced MRI sequences compared to MRI using STIR images and to analyze the association between these findings and disability and pain.

## Methods

### Patients

The patients were recruited from a previous randomized placebo-controlled trial involving 337 patients with knee OA according to the ACR (American College of Rheumatology) criteria [24] comparing five intra-articular injections of Hyalgan® and placebo, respectively [25]. Twenty-two patients with knee OA affecting the medial femoro-tibial articulation were randomly selected among 83 participants obtaining MRI at both baseline and follow-up. A list of these participants was sorted according to the day of birth, and the first 22 patients were chosen. Participants gave written informed consent prior to being enrolled in the study, including consent to publish study results and images [26]. The study was approved by the Central Denmark Region Committee on Biomedical and Research Ethics.

Patients filled out the Western Ontario and McMaster Universities Index (WOMAC) questionnaire at baseline and follow-up [27]. The WOMAC items, pain (5 items), stiffness (2 items) and physical function (17 items), were each scored using a 100 mm Visual Analogue scale with the following ranges: pain = 0–500, stiffness = 0–200, physical function = 0–1700, total score = 0–2400.

### Imaging

Radiography: Routine radiographic examination consisting of standing weight-bearing postero-anterior and lateral views with the knee in 30° flexion [28] were obtained in all patients at baseline.

MRI consisted of the following sequences: Sagittal STIR and sagittal and axial T1-weighted sequences before contrast. Following intravenous administration of Gadolinium contrast (Gd-DTPA, 0.2 mmol/ml, GE Healthcare AS, Oslo, Norway) using 0.1 mmol/kg with a maximum of 10 mmol DCE-MRI was obtained using a sagittal T1-weighted spoiled gradient echo sequence with four sagittal slices every 5 s with 50–65 repetitions, repetition time (TR) = 50.2 ms, echo time (TE) = 4.1 ms, field of view (FOV) = 16 cm, slice thickness (ST) = 4 mm, matrix = 128 × 128, flip angle 30° and acquisition time (AT) 4.2–5.4min. Finally, sagittal and axial T1 FS post-contrast sequences were performed using the following parameters: TR = 860 ms, TE = 20 ms, FOV = 16 cm, ST = 4.0 mm, interslice gap (IG) = 0.8 mm, matrix = 512 × 512, total AT 7.2min. DCE-MRIs were missing in 6 of 44 examinations for technical reasons and follow-up analyses could therefore not be performed in 5 patients.

Details of the MRI protocol are described in an additional file (see Additional file 1).

### Image analysis

All images were anonymized and separated before analysis. The radiographic changes were graded according to Ahlbäck [29] by an experienced musculoskeletal radiologist (NE) [30].

All MR sequences were assessed twice by a radiological registrar (FKN) with at least a 4-week interval between the assessments; all sequences except DCE-MRI were also assessed by NE. The assessments were preceded by training sessions amongst FKN, NE and AGJ of 15 examinations not included in the present study.

According to the definition of BMLs [10, 11] only changes directly adjacent to the subchondral bone were recorded and sections and areas of sections containing both bone marrow and extra-osseous soft tissue such as synovia were strictly excluded. Consequently, only three to four slices of the medial femoral condyle and tibial plateau remained for analyses. When four slices were available, the most medially located three slices were used for BML segmentation.

Images were analyzed for CEA-BMLs and STIR-BMLs by manual segmentation (MS) and computer assisted segmentation (CAS), respectively. Both methods have been tested on STIR images as described in a previous study [31] and all results of segmentation on STIR images have been presented in this publication. For a detailed method description of MS and CAS, please see Additional file 1.

DCE-MRI measurements were performed using Dynamika (DYNAMIKA software, version 3.2.6 (www.imageanalysis.org.uk, Leeds, UK)), a software performing a voxel-by-voxel-based line fit analysis to calculate parametric maps.

**Fig. 1:**
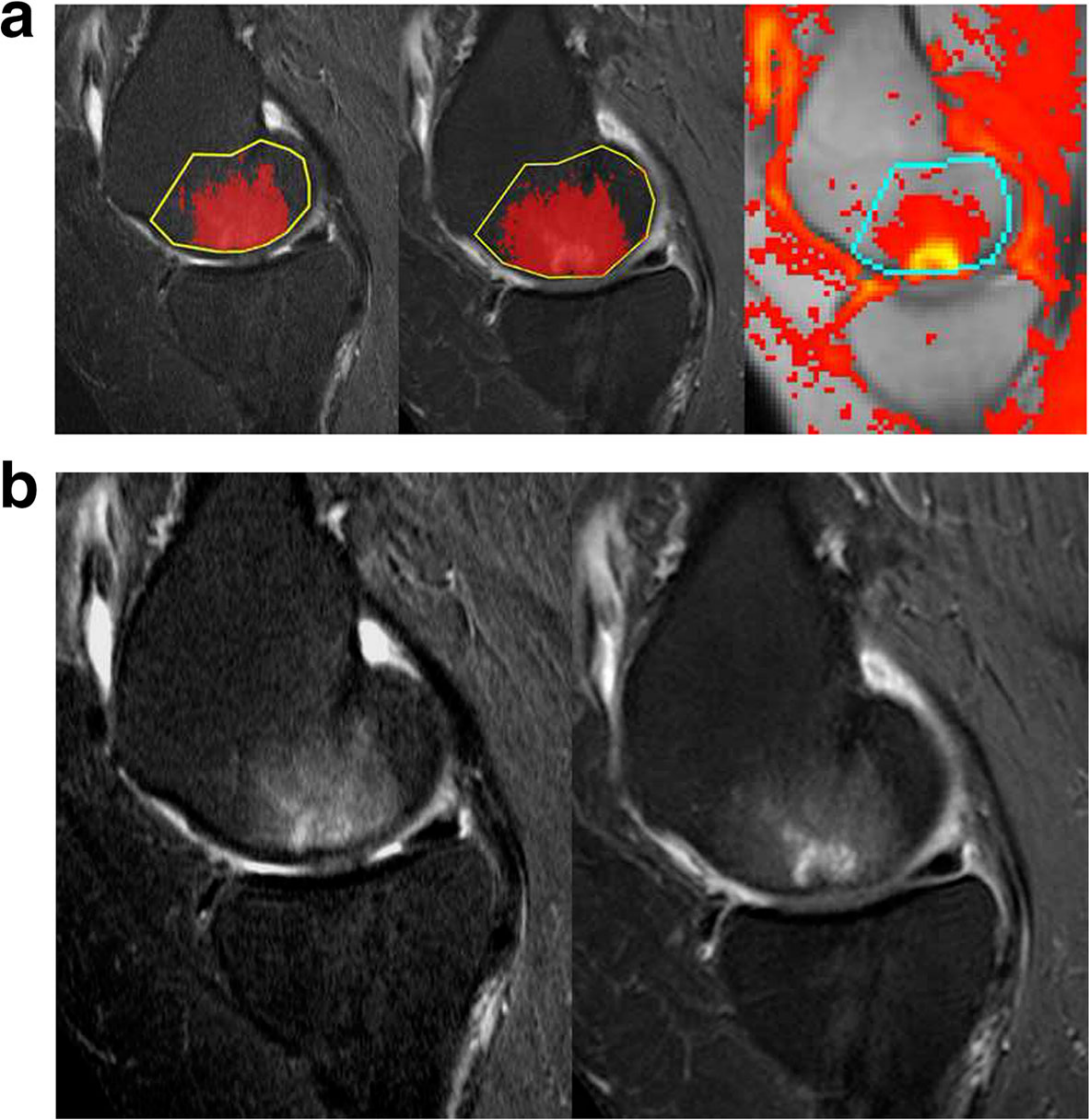
**a** Sagittal STIR (*left*), post-contrast T1 FS (T1 CE FS) (*middle*) and dynamic contrast enhanced MR images (DCE-MRI) (*right*) in one patient through the medial joint compartment with region of interests (ROIs) drawn in the weight-bearing femoral condyles. Computer assisted segmentation of BML on the STIR and T1 CE FS images is shown; the marked pixels represent areas above the threshold signal intensity, i.e., STIR-BML and CEA-BML, respectively. An area with rapid and steep enhancement (right image) is seen in the femoral condyle, *yellow* color. Pixels exhibiting a pathologic enhancement pattern within the ROI are visualized and segmented digitally. **b** Corresponding STIR (left) and T1 CE FS images (*right*) without ROI or pixel segmentation. (BML=bone marrow lesion, CEA-BML=contrast enhancing areas of bone marrow lesion)

One region of interest (ROI), outlined similar to CAS and MS (according to Hunter et al. [32]) (Fig. 1), was drawn in the medial femoral condyle and tibial plateau, respectively, to obtain quantitative data. Time intensity curves (TICs) expressing the enhancement pattern of each voxel were classified as one of four color-coded models for gadolinium uptake: (i) no enhancement; (ii) persistent enhancement; (iii) plateau (baseline → uptake → plateau); (iv) wash-out (baseline → uptake {→ plateau}→ wash-out) [23]. The volume of voxels with the pattern of ‘plateau’ and ‘wash-out’ enhancement were pooled to a value N_voxel_ and used as a measure of the volume of inflammation [22, 23]. The initial rate of enhancement (IRE) and maximal enhancement (ME) in the segmented area were also recorded to obtain the heuristic DCE variables IRExN_voxel_ and MExN_voxel_ [23]. Due to the applied method, no reference value was needed for calculating TICs.

### Statistical analysis

Data was analyzed using Analyse-it software (Analyse-it for Microsoft Excel (version 2.20) Ltd.; 2009). CEA-BML and STIR-BML were compared in all the 44 examined knees by Bland-Altman analyses, using plots, bias and confidence intervals (CIs) [33] and descriptively using median and quartiles after adjusting for the difference in inter-slice gap.

The inter- and intra-observer reliability were assessed based on all knee examinations irrespective of baseline or follow-up status by Bland-Altman analyses, using plots, bias and confidence intervals (CIs).

The WOMAC scores were analyzed descriptively using medians and ranges. The Spearman rho test was used to determine correlation coefficients between WOMAC scores and CEA-BML, STIR-BML and N_voxel_ parameters, respectively. Wilcoxon’s signed ranked test was used to compare median values between baseline and follow-up. The Spearman rho test and Wilcoxon’s signed ranked test were also performed after the segmentations of the femoral condyle and tibial plateau had been merged into one total knee score to express the total CEA-BML, STIR-BML and N_voxel_ involvement, respectively. A *p* value <0.05 was considered significant.

## Results

Patient characteristics are shown in Table 1.

**Table 1:** Baseline data of the patients

Patient characteristics (*n*=22)	
Age years, median (range)	61 (41–79)
Female gender (%)	18 (82%)
BMI, median (range)	25.7 (19.7–37.7)
Follow-up interval days, median (range)	334 (91–375)
Ahlbäck grading 0/1/2/3, No.	2/9/6/5

Inter- and intra-observer agreement for T1 CE FS and STIR as well as intra-observer agreement for DCE-MRI was high with small bias values (Table 2). Thus, the mean values of CEA-BML and STIR-BML measurements of the two assessors were subsequently used.

**Table 2:** Observer agreement for relative and absolute CEA-BML and STIR-BML measurements, and relative N_voxel_, irrespective of baseline/follow-up

Method	Area	Image sequence	Observer agreement	CEA-BML, STIR-BML and N_voxel_, relative			CEA-BML, STIR-BML and N_voxel_, absolute		
				Bias^a^,%	95% CI		Bias^a^, mm^3^/mm^2b^	95% CI	
MS	Femur	T1 CE FS	Inter-observer Intra-observer	−0.86 −0.98	−2.68 −2.37	0.95 0.42	−79 −94	−281 −199	122 11
	Tibia		Inter-observer Intra-observer	−0.67 −0.89	−4.2 −2.64	2.8 0.86	18 −58	−321 −147	358 31
	Femur	STIR	Inter-observer Intra-observer	−0.36 1.15	−1.45 −0.46	0.73 2.76	7 142	−129 −41	144 325
	Tibia		Inter-observer Intra-observer	−0.26 1.18	−0.82 −0.06	1.35 2.41	−83 129	−209 6	44 251
CAS	Femur	T1 CE FS	Inter-observer Intra-observer	0.23 −0.37	−1.22 −1.03	1.68 0.29	108 −71	−59 −185	276 43
	Tibia		Inter-observer Intra-observer	0.29 0.18	−1.57 −0.30	2.15 0.67	14 3	−94 −36	122 42
	Femur	STIR	Inter-observer Intra-observer	0.01 −0.10	−0.41 −0.42	0.43 0.21	15 67	−60 13	89 121
	Tibia		Inter-observer Intra-observer	−0.10 0.22	−0.70 −0.04	0.51 0.49	−74 61	−140 28	9 94
Dynamika	Femur Tibia	DCE-MRI	Intra-observer Intra-observer	−2.75 −2.5	−4.56 −5.37	−0.94 0.37	−27 −7	−50 −29	−5 15

^a^Bland-Altman analysis

^b^CEA-BML and STIR-BML in mm^3^, N_voxel_ in mm^2^

**Fig. 2:**
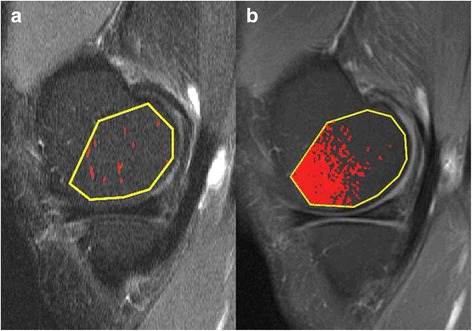
**a** Sagittal STIR and **b** T1 CE FS images through the medial condyle in a patient without STIR-BML or CEA-BML using MS. The marked pixels represent areas above the threshold signal intensity. **a** Random clusters of pixels are marked. The segmented area was 9 mm^2^ or 1.1%. **b** A large number of marked pixels are seen in the anterior part of the condyle. The segmented area was 280 mm^2^ or 33%

STIR-BMLs by MS and CAS and CEA-BMLs by MS according to the definition were not found in 13 femoral condyles and 15 tibial plateaus. Four of these femoral condyles and six tibial plateaus demonstrated CEA-BMLs by the CAS segmentation (Fig. 2). Voxels indicating inflammation by DCE-MRI were not seen in 5/38 femoral condyles and 5/38 tibial plateaus; STIR-BMLs by MS and CAS and CEA-BMLs by MS were not found in these examinations either, but CEA-BMLs by the CAS segmentation were seen in one femoral condyle and two tibial plateaus.

The average CEA-BML volumes using MS were smaller than the measured STIR-BML volumes with a negative bias of 3.2–3.5% for CEA-BML measurements compared to STIR-BML measurements (Table 3). However, the measured CEA-BML volumes were 4.1–5.3% larger than the STIR-BML volume using CAS (Table 3).

**Table 3:** Comparison of CEA-BML and STIR-BML measurements of all 44 examinations, relative and absolute values

Method	Area	CEA-BML% vs. STIR-BML%			CEA-BML vs. STIR-BML, absolute values, mm^3^		
		Bias^a^	95% CI		Bias^a^	95% CI	
MS	Femur	−3.52	−5.93	−1.12	−403	−651	−156
	Tibia	−3.23	−5.23	−1.22	−616	−860	−373
CAS	Femur	5.31	2.36	8.25	675	303	1046
	Tibia	4.11	1.72	6.50	237	34	474

^a^Bland-Altman analysis

The positive difference between CEA-BML and STIR-BML measurements using CAS (Table 4) was due to a small but general trend, as well as a number of outliers in the Bland-Altman plots. The 95% limits of agreement were exceeded in five CAS analyses of the femoral condyles (>3070 mm^3^) and in three analyses of the tibial plateaus. Four of the femoral condyle outliers were observed in two patients (baseline and follow-up); in one outlier patient, the high values were observed in both the femoral condyle and tibial plateau at follow-up. The remaining tibial outliers were observed in two different patients at baseline.

**Table 4:** Comparison of CEA-BML and STIR-BML volume in all 44 examinations, absolute values

Segmentation method	Area	Median CEA-BML and STIR-BML volume (25th - 75th quartile), mm^3^	
		T1 CE FS	STIR
MS	Femur	655 (0–2435)	812 (0–3203)
	Tibia	439 (0–1848)	942 (0–3032)
CAS	Femur	1281 (251–3715)	568 (150–2399)
	Tibia	597 (207–2633)	295 (137–1591)
CAS^a^	Femur	881 (199–2614)^a^	N.A.
	Tibia	384 (195–2082)^a^	N.A.

*N.A.* not applicable

^a^Outliers excluded

**Fig. 3:**
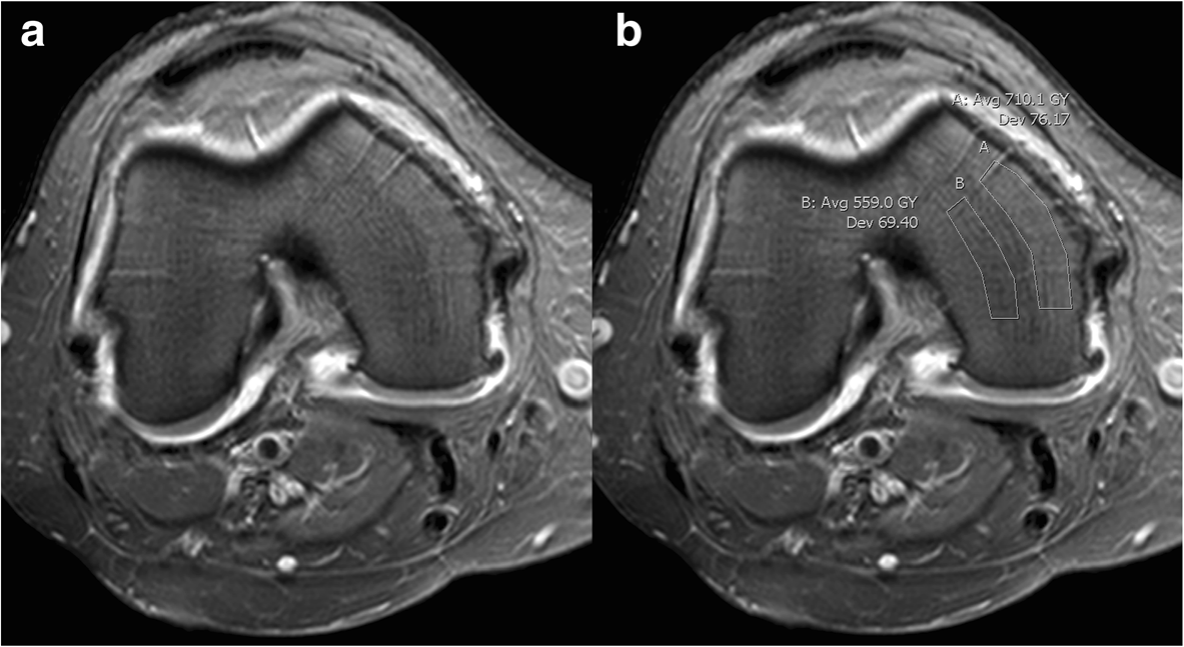
Trans-axial post-contrast T1 FS MR images of the femoral condyles. Two identical images without (**a**) and with (**b**) calculations of Gray (GY), performed in Impax. The difference in signal intensity is barely visible in image (**a**) between the medial and central (lateral) part of the medial femoral condyle. Yet, there is a difference in GY between the areas of 151.1 GY, meaning the average GY in the central portion is ~ 21% lower than in the medial portion. Also note the blood vessels perforating the condyles from the periphery

The outliers were analyzed in a consensus reading between NE and FKN and revealed an apparent increase in signal intensity in a superficial rim of the medial and lateral femoral condyles (Figs. 2 and 3) and, to a smaller extent, the tibial plateaus. Exclusion of the outliers narrowed the variation (Table 4) and they were not included in the analyses regarding association with disability and pain, nor with changes during follow-up.

Analyses of DCE-MRI revealed a median relative/absolute N_voxel_ distribution for all examinations of 27.4% (range 0.0–93.2%)/284 mm^2^ (range 0–1077 mm^2^) and 24.0% (range 0.0–94.8%)/218 mm^2^ (range 0–786 mm^2^) in the femoral condyles and tibial plateaus, respectively. Since DCE-MRI measurements were confined to one slice, the results were not directly compared to the data from the static MR sequences.

### Clinical and radiological correlation

A significant correlation was seen between the total volume of CEA-BML and STIR-BML in the medial joint compartment and WOMAC_pain_ and WOMAC_total_ scores using MS and CAS (Table 5). Both WOMAC_pain_ and WOMAC_total_ scores were significantly correlated with N_voxel_ and MExN_voxel_, but not with IRExN_voxel_. The ME value showed very little variation (femur: median 1.11, range 1.04–1.56; tibia: median 1.07, range 1.03–1.48) in the 33 examinations with postitive N_voxel_ findings and the addition of this value did not seem to add any significant information. The corresponding IRE values were very small (femur: median 0.001, range 0.000–0.006; tibia: median 0.001, range 0.000–0.005) and they only seemed to distort the correlations (Table 5).

**Table 5:** Comparison of WOMAC scores with CEA-BML, STIR-BML and N_voxel_–parameters

Method	Segmentation parameter	WOMAC - pain				WOMAC - total			
		Correlation coefficient^a^	95% CI		2-tailed p	Correlation coefficient^a^	95% CI		2-tailed p
MS	CEA-BML	0.47	0.20	0.67	0.001	0.49	0.23	0.69	0.001
	BML	0.46	0.19	0.67	0.002	0.49	0.23	0.69	0.001
CAS	^b^CEA-BML	0.40	0.12	0.63	0.008	0.37	0.08	0.61	0.014
	BML	0.37	0.09	0.60	0.012	0.39	0.11	0.62	0.009
Dynamika	N_voxel_	0.37	0.06	0.62	0.022	0.44	0.14	0.66	0.006
	ME x N_voxel_	0.39	0.08	0.63	0.016	0.45	0.15	0.67	0.005
	IRE x N_voxel_	0.20	−0.12	0.49	0.22	0.28	−0.04	0.55	0.086

Data was analyzed using imaging data from the entire medial knee joint, irrespective of baseline/follow-up. BML and N_voxel_ were calculated based on the relative involvement. MExN_voxel_ and IRExN_voxel_ were calculated by multiplying the absolute number of N_voxel_ by ME and IRE, respectively

^a^Spearman’s rank correlation data

^b^5 femoral and 3 tibial outliers excluded

There was a significant reduction in total WOMAC score of 286 (*p* ≤ 0.023) from baseline to follow-up. No other significant changes were observed (Table 6).

**Table 6:** Median differences in change of WOMAC scores and imaging parameters from baseline to follow-up

Method	Segmentation parameter	Baseline, median (range)	Follow-up, median (range)	*p*-values*
WOMAC_total_	N.A.	794 (76–1444)	234 (9–1782	0.023
WOMAC_pain_	N.A.	140 (18–311)	44 (3–373)	0.054
MS	CEA-BML	6.5 (0–67.0)	7.5 (0–79.4)	0.21
	BML	8.1 (0–70.5)	11.4 (0–86.8)	0.71
CAS	CEA-BML^a^	8.1 (0.1–44.9)	8.6 (1.0–81.3)	0.18
	BML	5.6 (0.1–39.8)	5.3 (0.6–79.8)	0.97
Dynamika	N_voxel_	29.3 (0.0–99.3)	18.8 (0.0–86.1)	0.13
	MExN_voxel_	379.4 (0.0–1442.1)	281.6 (0.0–1679.5)	0.13
	IRExN_voxel_	0.2 (0.0–3.3)	0.2 (0.0–4.1)	0.45

Data was analyzed using imaging data from the entire medial knee joint. CEA-BML, BML and N_voxel_ were calculated based on the relative involvement. MExN_voxel_ and IRExN_voxel_ were calculated by multiplying the absolute number of N_voxel_ by ME and IRE, respectively

*N.A.* not applicable

*Changes from baseline to follow-up, Wilcoxon’s signed rank test

^a^5 femoral and 3 tibial outliers excluded

## Discussion

Our study showed that the measured volume of CEA-BMLs on average were smaller than the measured STIR-BML volumes using MS, but CAS measurements of CEA-BMLs were not consistently reliable due to signal disturbances in some of the T1 CE FS sequences. There was a positive correlation between CEA-BML/STIR-BML size and pain but no significant correlation between change in CEA-BML/STIR-BML and WOMAC_pain_ during follow-up.

Contrast enhanced MRI is not routinely used in OA studies, and the enhancement characteristics have only been evaluated in a few studies [11, 16, 17]. These studies have focused on size measurements of BMLs, comparing fluid sensitive sequences, i.e., PD FS or STIR, with contrast enhanced T1 FS series; only one study [11] has, however, focused specifically on OA related BMLs. In this study the pathological areas on T1 CE FS were generally smaller than BML-areas on PD FS [11]. In the studies by Schmid et al. and Mayerhoefer et al., only a minor number of patients had “bone marrow edema” due to OA (3.5and 26.7%, respectively), other reasons being osteonecrosis, bone bruise and osteochondral lesions [16, 17]. Schmid et al. found that the volume of bone marrow edema was slightly larger by STIR, whereas Mayerhoefer et al. found that the volume was largest on T1 CE FS using a gadopentate dose of 0.1 mmol/kg but slightly smaller on T1 CE FS than on STIR using a gadopentate dose of 0.075 mmol/kg [16, 17].

In line with the study by Roemer et al. [11], we found that the CEA-BML volume by T1 CE FS generally was smaller than the BML volume by STIR using MS. We did, however, find CAS to be unreliable on T1 CE FS sequences in some cases where areas of increased signal intensity were seen in the peripheral parts of the femoral condyles and tibial plateaus (Fig. 2). These signal disturbances could be due to the anatomical composition of the knee (Fig. 3), non-uniform fat saturation, magnetic field inhomogeneity, receive coil inhomogeneity or a combination hereof. Since our threshold calculation was performed using the most central slices in the lateral femoral condyle and tibial plateau, the signal disturbances did not affect the threshold calculation.

We found a positive correlation between CEA-BML/STIR-BML volume and symptoms and also between N_voxel_ and MExN_voxel_ and symptoms. However, there was no indication that CEA-BMLs or the dynamic parameters N_voxel_ or MExN_voxel_ were better correlated with pain than STIR-BMLs. Thus, the results cannot support that changes in vascularization in BMLs play a role in the symptomatology of BML. Therefore, the use of contrast agents for visualization of BMLs in OA seems unnecessary in clinical practice.

The median scores for pain decreased during follow-up but it was not accompanied by a corresponding decrease of BMLs by MRI. This indicates that other knee joint changes are of importance. Consistent with this, other factors have been reported to be associated with OA symptoms [5, 23].

Our study has a number of limitations, especially the small number of participants. We only analyzed subchondral bone marrow changes and did not look for other pathologies known to be correlated with pain, e.g., synovitis or meniscal damage [23]. There could be anatomical and technical reasons for the occurrence of altered signal intensity by CEA-BML, which was not analyzed further. In our material, this was evident both as a small general trend and in a minor number of considerable outliers on the T1 CE FS-sequences, underlining both the need for high quality images and the fact that images cannot solely be analyzed by computer software.

The strength of our study is the use of an exact definition of the slices and areas used for segmentation. By excluding sections with partial volume artifacts from the surrounding soft tissue, our data was not distorted by signal intensity changes from the synovia and/or joint fluid. We have not found similar restrictions of ROI definitions in other OA analyses of BMLs [10, 32, 34–36]. In the illustrations of the scoring system proposed by the Canadian CareArthritis [36], areas affected by partial volume from synovitis seems to be included in the registration of BMLs.

## Conclusions

In conclusion, we found that contrast enhancing areas of BMLs on average were smaller than STIR-BMLs although the differences were small and that manual segmentation may be needed to obtain valid CEA-BML volumes. The CAS method proved suitable for BML segmentation on fluid sensitive sequences being quickly performed and reproducible. Both CEA-BMLs and STIR-BMLs were similarly correlated to symptoms. The volume of voxels indicating inflammation by the DCE-MRI sequence were equally correlated to symptoms.

## Additional file

Additional file 1: Supplementary imaging protocol, method description and figures. (ZIP 5644 kb)

## Abbreviations

BML: Bone marrow lesion
CAS: Computer assisted segmentation
CE: Contrast enhanced
CEA-BML: Contrast enhancing area of bone marrow lesion
CI: Confidence interval
DCE-MRI: Dynamic contrast enhanced-MRI
IRE: Initial rate of enhancement
ME: Maximum enhancement
MS: Manual segmentation
OA: Osteoarthritis
PD: Proton density
ROI: Region of interest
TIC: Time intensity curve
WOMAC: Western Ontario and McMaster Universities Index
