# Supplementary material for: Assessment of subchondral bone marrow lesions in knee osteoarthritis by MRI: a comparison of fluid sensitive and contrast enhanced sequences
Source: BMC Musculoskelet Disord. 2016 Nov 16;17:479. doi: 10.1186/s12891-016-1336-9 (PMC5112734; doi:10.1186/s12891-016-1336-9)
Supplement: Additional file 1: — Supplementary imaging protocol, method description and figures. (ZIP 5644 kb) [file 12891_2016_1336_MOESM1_ESM.zip › Additional file, submitted minus feltkoderR2.docx]

**Additional file**

**Assessment of subchondral bone marrow lesions in knee osteoarthritis by MRI: a comparison of fluid sensitive and contrast enhanced sequences**

Flemming K. Nielsen, Anne Grethe Jurik, Anette Jørgensen, David A. Petersen, Niels Egund

**Supplementary content**

- MR imaging protocol

- Image analysis

**Supplementary references**

**Supplementary figures**

- Figure A1

**MR imaging protocol**

MR examinations were performed using a 1.5 Tesla system (Vision, Siemens, Erlangen, Germany) and a transmit receive four-channel knee coil. The baseline and follow-up examinations consisted of the following sequences: Sagittal STIR, repetition time (TR) = 5000 ms, echo time (TE) = 29 ms, inversion time = 150 ms, field of view (FOV) = 20 cm, slice thickness (ST) = 4.0 mm, interslice gap (IG) = 0.4 mm, matrix = 266 × 512 pixels, one excitation, and acquisition time (AT) 5.3 min and sagittal and axial T1-weighted sequences. Gadolinium contrast (Gd-DTPA, 0.2 mmol/ml, GE Healthcare AS, Oslo, Norway) was injected at a peripheral intravenous site (0.1 mmol/kg with a maximum of 10 mmol) using a power injector followed by a saline flush. DCE-MRI was performed using a sagittal T1-weighted spoiled gradient echo sequence with four sagittal slices every 5 seconds with 50 - 65 repetitions, TR = 50.2 ms, TE = 4.1 ms, FOV = 16 cm, ST = 4 mm, matrix = 128 x 128, flip angle 30° and AT 4.2-5.4 min. Finally, sagittal and axial T1 FS post contrast sequences were performed using the following parameters: TR = 860 ms, TE = 20 ms, FOV = 16 cm, ST = 4.0 mm, IG = 0.8 mm, matrix = 512 × 512 pixels, one excitation and total AT 7.2 min. Sagittal STIR and T1 CE FS images were obtained perpendicular to the line connecting the dorsal aspect of the medial and lateral femoral condyle (Figure A1). The four sagittal slices in the DCE-MRI series were centered with two slices in the central medial and two slices in the central lateral femoro-tibial compartment (Figure A1). Only the sagittal STIR, sagittal and axial T1 CE FS and DCE-MR images were analyzed in the present study. DCE-MR images were missing in 6 of 44 examinations for technical reasons.

**Image analysis**

CEA-BMLs and STIR-BMLs were analyzed by manual segmentation (MS) and computer assisted segmentation (CAS). The segmentations were confined to the posterior 2/3 of the femoral condyles according to Hunter et al. [1] and to the tibial plateaus, demarcated corresponding to a straight line 20 mm distal to the lowest point of the subchondral bone. Only the three central slices medially were used (Figure A1). Laterally, one slice using MS or two slices using CAS were used for calculation of signal intensity threshold in the femoral condyle and tibial plateau, respectively. The threshold by MS was defined as the average signal intensity (SI) plus one standard deviation (SD) and by CAS as the average SI plus two SDs, in accordance with our previous analyses [2].

MS was performed by manually outlining CEA-BMLs and STIR-BMLs using a standard radiological workstation (Agfa Impax, Mortsel, Belgium, version 6.3.1.8000) with 2K Bracco screens and standard graphic tools. As guidance for CEA-BML/STIR-BML demarcation, a rectangular region of interest of 5 mm^2^ was used to identify areas with SIs above and below the SI threshold.

CAS evaluations were performed using a MATLAB (the MathWorks, Sweden) graphic user interface developed by one of the authors (DP) and is a method based on pixel thresholding: voxels in the medial femoral condyle and tibial plateau with SIs above the threshold being calculated digitally, yielding the relative and absolute CEA-BML/STIR-BML volumes.

References

1. Hunter DJ, Lo GH, Gale D, Grainger AJ, Guermazi A, Conaghan PG. The reliability of a new scoring system for knee osteoarthritis MRI and the validity of bone marrow lesion assessment: BLOKS (Boston Leeds Osteoarthritis Knee Score). Ann Rheum Dis. 2008;67:206-211.

2. Nielsen FK, Egund N, Peters D, Jurik AG. Measurement of bone marrow lesions by MR imaging in knee osteoarthritis using quantitative segmentation methods--a reliability and sensitivity to change analysis. BMC Musculoskelet Disord. 2014;15:447.

Figure legend

**Figure A1**

(a) Trans-axial post-contrast T1 FS MR images of the femoral condyles illustrating the orientation of the sagittal T1 CE FS and STIR sequences and (b) the sagittal DCE-MR sequence. (a) The sagittal slices by T1 CE FS and STIR were orientated perpendicular to a line joining the dorsal aspect of the femoral condyles. The white lines in the medial condyle illustrate slices with potential partial volume, leaving three slices suitable for CEA-BML/STIR-BML evaluation. Laterally, ≥ 2 slices were generally available for reference calculation. (b) The two slices by DCE-MRI were centered along the condyles. The slice located most centrally in the medial compartment was used to evaluate dynamic contrast enhancement.
