# Supplementary figures and images for: Assessment of subchondral bone marrow lesions in knee osteoarthritis by MRI: a comparison of fluid sensitive and contrast enhanced sequences
Source: BMC Musculoskelet Disord. 2016 Nov 16;17:479. doi: 10.1186/s12891-016-1336-9 (PMC5112734; doi:10.1186/s12891-016-1336-9)

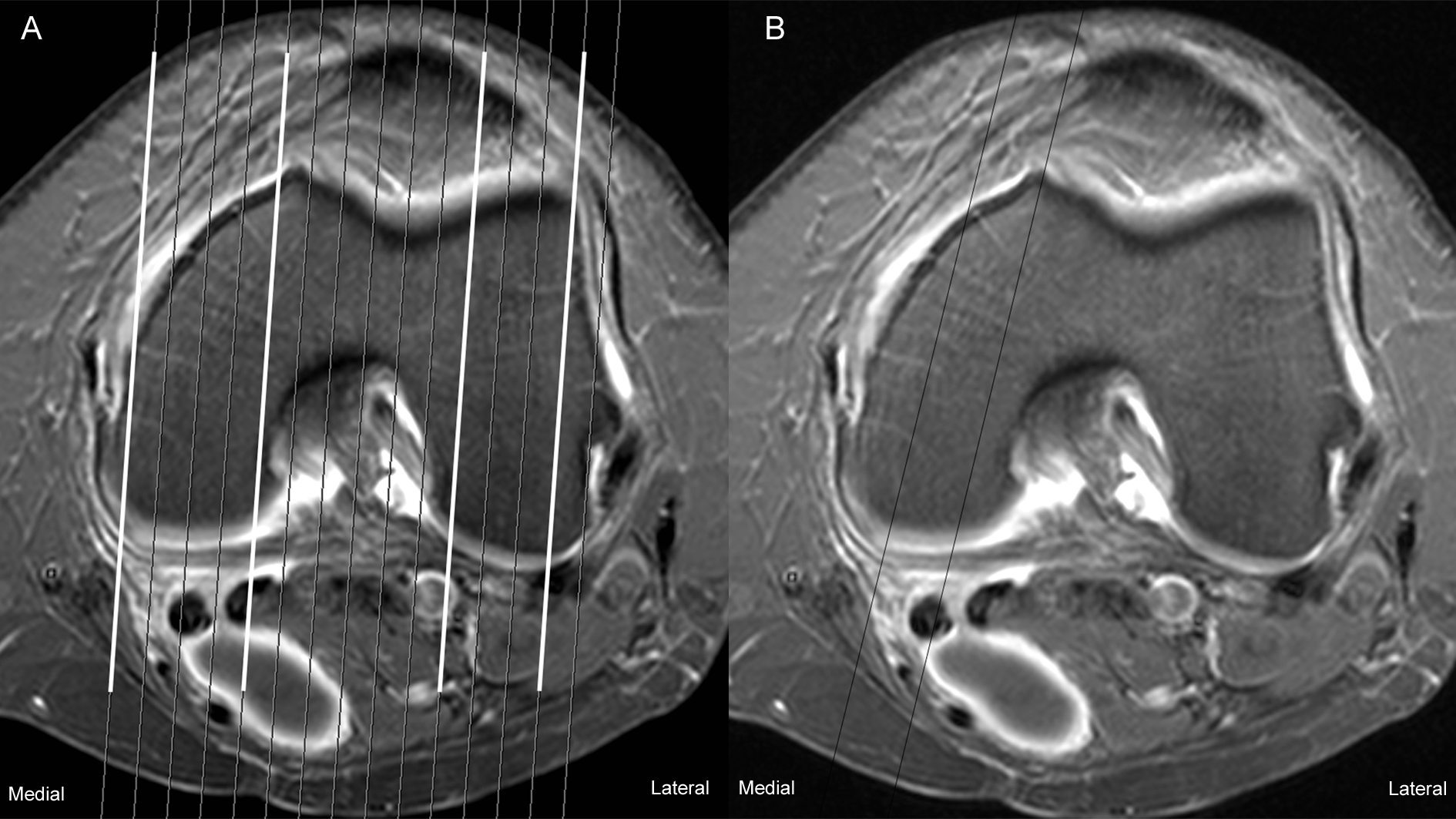

Supplement: Additional file 1: — Supplementary imaging protocol, method description and figures. (ZIP 5644 kb) [file 12891_2016_1336_MOESM1_ESM.zip › Figure A1R2.tif]
